# Vedolizumab use after failure of TNF-α antagonists in children and adolescents with inflammatory bowel disease

**DOI:** 10.1186/s12876-018-0868-x

**Published:** 2018-09-15

**Authors:** Anna-Maria Schneider, Daniel Weghuber, Benjamin Hetzer, Andreas Entenmann, Thomas Müller, Georg Zimmermann, Sebastian Schütz, Wolf-Dietrich Huber, Judith Pichler

**Affiliations:** 1 Department of Pediatrics, Paracelsus Medical University, Salzburg, Austria; 2 Departments of Pediatrics, Innsbruck Medical University, Anichstrasse 35, 6020 Innsbruck, Austria; 3 Department of Mathematics, Paris Lodron University, Salzburg, Austria; 4 Spinal Cord Injury and Tissue Regeneration Centre Salzburg, Paracelsus Medical University, Salzburg, Austria; 5 Department of Pediatric Nephrology and Gastroenterology, Medical University Vienna, Vienna, Austria

**Keywords:** Crohn’s disease, Ulcerative colitis, Paediatrics, Integrin antagonist

## Abstract

**Background:**

Vedolizumab is safe and effective in adult patients with Crohn’s disease (CD) and ulcerative colitis (UC); however, data in children with inflammatory bowel disease (IBD) are scarce. Therefore, we evaluated vedolizumab use in a cohort of Austrian paediatric patients with IBD.

**Methods:**

Twelve patients (7 female; 7 CD; 5 UC), aged 8–17 years (median, 15 years), with severe IBD who received vedolizumab after tumour necrosis factor α antagonist treatment were retrospectively analysed. Clinical activity scores, relevant laboratory parameters, and auxological measures were obtained at infusion visits.

**Results:**

In the CD group, 1/7 patient discontinued therapy due to a severe systemic allergic reaction; 1/7 and 2/7 patients achieved complete and partial response, respectively, at week 14; and 3/7 patients discontinued therapy due to a primary non-response or loss of response. In the UC group, complete clinical remission was achieved at weeks 2, 6, and 14 in 2/5, 1/5 and 1/5 patients respectively; partial response was observed in one patient at week 2. CD activity scores did not significantly change from baseline to week 38 (median 47.5 vs. 40 points, *p* = 1,0), while median UC activity scores changed from 70 to 5 points (*p* < 0,001). Substantial weight gain and increased albumin and haemoglobin levels were observed in both groups.

**Conclusion:**

These results demonstrate that vedolizumab can be an effective treatment for individual paediatric patients with IBD who are unresponsive, intolerant, or experience a loss of efficacy in other therapies. However, vedolizumab appears to be more effective in paediatric UC than in paediatric CD.

## Background

Inflammatory bowel disease (IBD), including Crohn’s disease (CD), ulcerative colitis (UC), and unclassified IBD (IBD-U), is a multifactorial disease that results from an intricate interplay between genetic predisposition, altered immune response, changes in the intestinal microbiota, and environmental factors. Together, these factors contribute to the destruction of the intestinal epithelial barrier, increased gut permeability, and an influx of immune cells [1, 2].

Treatment consists of induction, maintenance therapy and, during a flare, intensified therapy, with the aim of achieving and maintaining clinical remission. In paediatric CD, but not UC, exclusive enteral nutrition therapy is a first-line treatment and is as effective as corticosteroids, with less side effects [3]. Persistent unsatisfactory results from treatment with immuno-modulators (such as azathioprine or 6-mercaptopurine) or mesalazine and corticosteroids have led to the development of biologicals in the treatment of IBD. The most common biologicals, infliximab and adalimumab, work by inhibiting the proinflammatory cytokine, tumour necrosis factor alpha (TNF- α), and are effective in moderate to severe paediatric CD and UC [4, 5]. Unfortunately, up to a third of patients do not have a primary response, and primary responders may develop a loss of efficacy [6]. Patients who are switched to a second anti-TNF- α drug are less likely to show a good response and may develop antibodies [7].

Several novel agents are currently being investigated, including vedolizumab, golimumab, and other biologicals [8]. Vedolizumab is a humanized α4β7-integrin antagonist, characterized by a gut-selective mechanism of action. By binding to surface-expressed α4β7 integrin, it inhibits T-lymphocyte migration into inflamed intestinal tissue. Its efficacy and safety has been evaluated in adult patients with moderate to severe UC and CD [9–12]. Initial data suggest that vedolizumab exerts its full effect after 14 weeks. Although vedolizumab was not more effective than placebo after prior TNF- α antagonist failure in adult patients with CD at week 6 (remission rate, 15.2%), a higher proportion of remission was achieved with vedolizumab than with placebo at week 10 [13]. Experience with vedolizumab in paediatric patients is limited, as its use is off-label, when available conventional therapy (TNF- α antagonists or immuno-modulators) has failed. Currently, only two case series from the United States and one from Europe have been published. These studies indicate a clinical benefit of vedolizumab in the induction and maintenance of remission in paediatric IBD, with a very good safety profile [14–16].

The aim of this study is to complement the limited existing data by reporting the efficacy and safety of vedolizumab in a cohort of Austrian paediatric patients with IBD.

## Methods

This study was approved by the local ethics committee *(No 1140/2017)* and was conducted in accordance with the Helsinki declaration.

### Study design and patient population

The present retrospective case series comprised patients from three tertiary paediatric care centres in Austria. In total, 12 paediatric patients with severe IBD (CD, *n* = 7; UC, *n* = 4; IBD-U, *n* = 1) were started on vedolizumab (Entyvio®, Takeda, Osaka, Japan) between November 2014 and August 2016, resulting in an observational period of at least 38 weeks (mean duration, 52 weeks; range, 38–96 weeks).

The indication for initiating vedolizumab treatment was a severe disease course, with steroid dependency and failure or intolerance of TNF-α antagonists, including infliximab, adalimumab, and golimumab. All patients were started on vedolizumab using a dosing regimen of 6 mg/kg, with a maximum dosage of 300 mg iv. Induction therapy was administered in weeks 0, 2, and 6, followed by maintenance infusions every 4–8 weeks (depending on the disease course).

At every vedolizumab infusion visit, the short paediatric Crohn’s disease activity index (shPCDAI) or paediatric ulcerative colitis activity index (PUCAI) was completed, as appropriate for the diagnosis [17, 18]. In addition, relevant laboratory values (C-reactive protein [CRP], haemoglobin [HB], haematocrit [HKT], and albumin) and auxological measures (height and weight) were obtained. Fecal calprotectin in UC was obtained at baseline and week 38.

Primary outcomes comprised the clinical response at weeks 2, 6, 14, 22, and 38. Secondary outcomes included changes in weight, height, and inflammatory marker levels. Safety and adverse events were documented.

### shPCDAI and PUCAI

As the erythrocyte sedimentation rate (ESR) was not available at every visit, the shPCDAI index was used [17]. The shPCDAI index was created by retaining and re-weighting the components of the PCDAI, and assesses general well-being, abdominal pain, weight, stools, palpation of the abdomen, and extra-intestinal manifestations. The shPCDAI has a maximum score of 90 points; remission is defined as a score < 10 points, and scores of 10–25 points, > 25 points, and > 40 points reflect mild, moderate, and severe disease, respectively.

The PUCAI score reflects disease activity in paediatric UC and has a maximum score of 85 points. Remission is defined as a score < 10 points, and scores of 10–34 points, 35–64 points, and > 65 points reflect mild, moderate, and severe disease, respectively [18].

### Statistics

Due to the small sample size and the ordinal scale of the data, nonparametric ANOVA-type statistics were used to compare baseline and follow-up scores, and the corresponding effect measure (Relative Treatment Effect [RTE]) was calculated. Calculating the RTE is useful in studies with a small sample size or scores that are composed of several items, like the shPCDAI and PUCAI [19]. An RTE > 0.5 indicates a tendency towards increased scores, whereas an RTE < 0.5 indicates a tendency towards decreased scores at a certain time point relative to the value at baseline [20]. All *p* values were corrected using Bonferroni’s adjustment for multiple testing. Adjustments were performed separately for the CD and UC groups. Secondary outcomes were given as median and range. All calculations were performed using R software (version, 3.3.2; R Core Team 2016).

## Results

The median age of the 12 enrolled patients at baseline was 15 years (range, 8–17 years). All patients were previously treated with infliximab, 11 patients (92%) were previously treated as well with adalimumab, and one patient (8%) was previously treated with golimumab as a third biologic agent. The main reason for medication discontinuation was loss of response (58% for infliximab, 64% for adalimumab). Demographic data at start of vedolizumab treatment are shown in Table 1. Among the 7 patients with CD, one had neurofibromatosis type I as a second underlying disease, one showed extra-intestinal manifestations (arthritis), and one had a perirectal fistula. None of the patients underwent any surgery prior to vedolizumab treatment; however, one patient with UC underwent total colectomy after 60 weeks of treatment.

**Table 1:** Patient characteristics at vedolizumab start

*Total n (%)*	12 (100)
*Male n (%)*	5 (42)
*Type of disease n (%)*	
*Crohn’s disease*	7 (58)
*Ulcerative colitis*	4 (34)
*Unclassified inflammatory bowel disease*	1 (8)
*Age yr, median (range)*	15 (8–17)
*Disease duration yr, median (range)*	2 (1–9)
*Previous biologicals n (%)*	
*Infliximab*	12 (100)
*Adalimumab*	11 (92)
*Golimumab*	1 (8)
*Reasons for discontinuation*	
*Infliximab n (%)*	
*Primary non-responder*	3 (25)
*Loss of response*	7 (58)
*Anaphylactic reaction*	2 (17)
*Adalimumab n (%)*	
*Primary non-responder*	2 (18)
*Loss of response*	7 (64)
*Local allergic reaction*	2 (18)
*Golimumab n (%)*	
*Loss of respone*	1 (100)

One patient discontinued therapy due to a severe general systemic allergic reaction at the second infusion of vedolizumab. This patient was included in the demographic data and short individual description in Tables 1 and 3, but was excluded in further investigations and outcome data (eg. Table 2). All of the remaining 11 patients received at least 5 vedolizumab infusions and completed 22 weeks of surveillance. Total observation and evaluation of the clinical course was maintained until week 38.

**Table 2:** Anthropometry, lab values and concomitant medication at baseline and week 38, median (range)

	Baseline	Week 38
*Weight, kg (z score for age)*		
*Crohn’s disease (CD)*	44.4 (1.05)	48 (1.26)
*Ulcerative colitis (UC)*	34.3 (1.36)	44 (1.86)
*Height, cm*		
*CD*	164.0 (138–172)	164.2 (140–172)
*UC*	146 (127–155)	148 (130–155.5)
*Haemoglobin, g/dl*		
*CD*	8.4 (6.9–13.0)	10.5 (7.8–12.3)
*UC*	10.4 (8.0–12,3)	12.1 (8.0–14.8)
*Haematocrit, %*		
*CD*	26.2 (22.9–40.1)	32.7 (25–38.3)
*UC*	29.6 (25.0–37.0)	35.8 (25–41.1)
*Albumin, g/l*		
*CD*	29.6 (18.0–37.5)	35.0 (28.1–42.4)
*UC*	27.9 (25.6–41.0)	40 (36.5–48.0)
*C- reactive protein, mg/dl*		
*CD*	4.0 (3.0–8.1)	2.6 (1.7–4.56)
*UC*	1.1 (0.1–3.0)	0.9 (0.1–2.6)
*fecal calprotectin, μg/g stool*		
*CD*	not applicable (n/a)	n/a
*UC*	1482 (84–5910)	187 (5–9750)
*Concomitant medication*		
*Corticosteroids*		
*CD, n*	4/6	4/6
*dosage, milligram per kilogram bodyweight (mg/KG BW)*	0.25 (0.1–0.4)	0.23 (0.1–0.4)
*UC, n*	4/5	1/5
*dosage, mg/KG BW*	1 (0.5–1.5)	0.2
*Azathioprine*		
*CD, n*	2/6	2/6
*UC, n*	1/5	1/5
*Mesalazine*		
*CD, n*	2/6	2/6
*UC, n*	0/5	1/5

### Clinical response

In the CD cohort, 1/6 patients reached complete remission and 2/6 had partial response at week 14. Three patients discontinued treatment due to a primary non-response in week 22 or a loss of response in weeks 34 and 37. In the UC cohort, complete clinical remission was reached in 2/5 patients at week 2, 1/5 patients at week 6, and 1/5 patients at week 14; partial response was observed in one patient at week 2. At the last follow-up, one patient with UC underwent a total colectomy 60 weeks after the first vedolizumab infusion due to a loss of response, steroid dependency, and severe disease course. The shPCDAI did not significantly change from baseline to week 38 (median 47.5 vs. 40 points, *p* = 1,0), while median PUCAI changed from 70 to 5 points (*p* < 0,001).

**Fig. 1:**
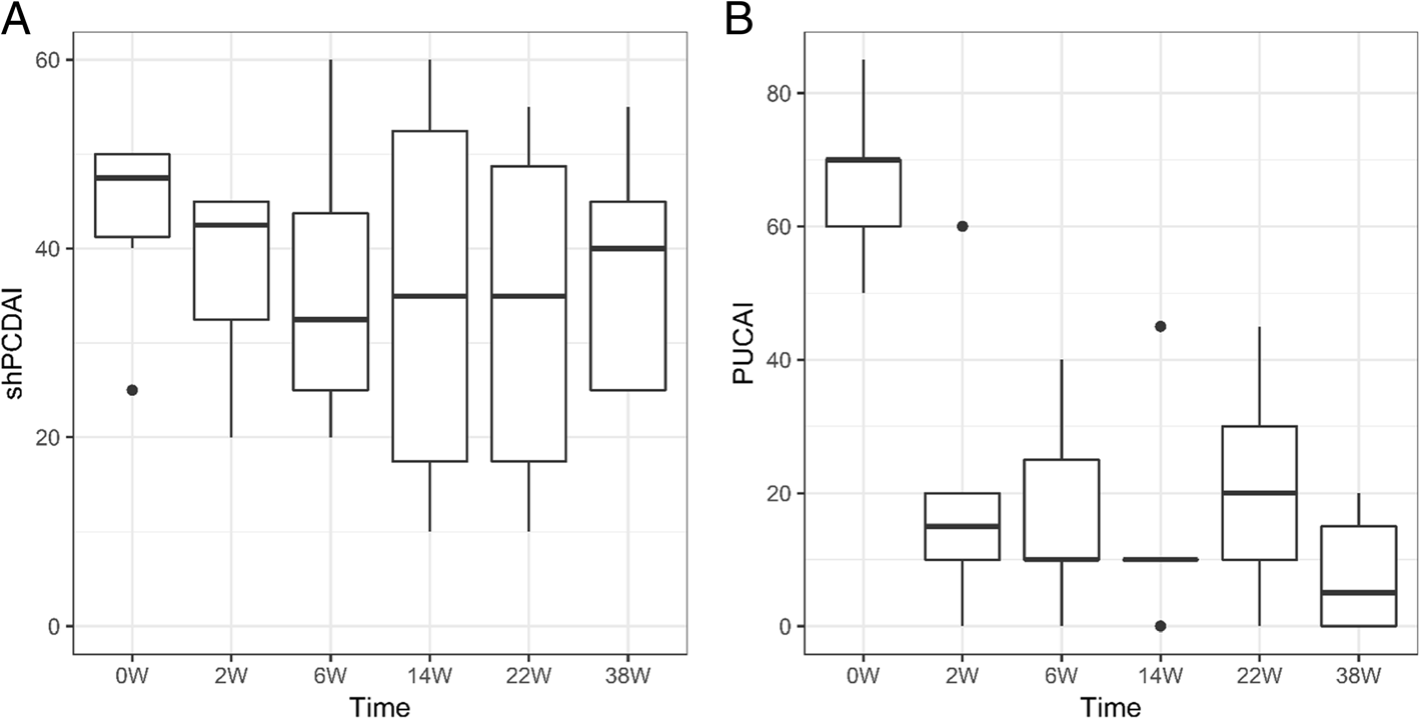
Clinical activity scores using the short paediatric Crohn’s disease activity index (shPCDAI) in the CD cohort (**a**) and the paediatric ulcerative colitis activity index (PUCAI) in the UC cohort (**b**). The dots indicate observations, which exceed the third quartile (i.e., the upper boundary of the box) by more than 1.5 times the interquartile range

**Fig. 2:**
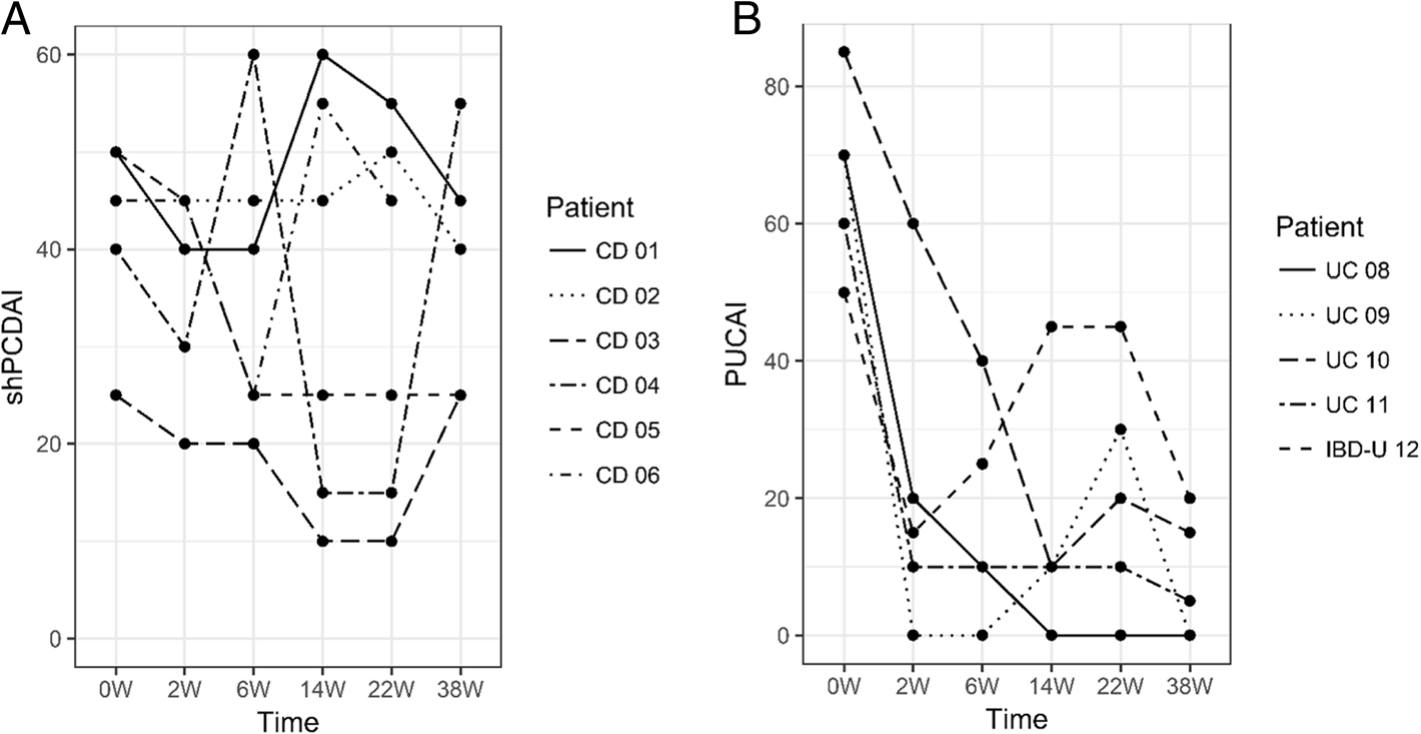
Individual courses of clinical activity scores using the short paediatric Crohn’s disease activity index (shPCDAI) in the CD cohort (**a**) and the paediatric ulcerative colitis activity index (PUCAI) in the UC cohort (**b**)

The clinical activity scores for each group and the individual courses are shown in Figs. 1 and 2. One patient in the CD group was excluded in the comparison between baseline and week 38 scores due to missing data. Table 3 provides an overview of the dosage, therapy duration, and brief information regarding the individual clinical courses until the last follow-up.

**Table 3:** Vedolizumab therapy in 12 patients with IBD

ID	Dosage in mg	Duration of therapy at last follow up, weeks	Response and outcome of therapy
CD01	250	36	Loss of response, discontinuation in week 37
CD02	300	64	No remission achieved, dosing interval 4 weeks, variable disease course, therapy ongoing
CD03	300	64	Remission after 14 weeks, variable disease course, therapy ongoing
CD04	300	36	Partial remission in week 14, loss of response, discontinuation in week 34
CD05	300	60	No remission achieved, variable disease course, therapy ongoing
CD06	200	16	Primary non-responder, discontinuation in week 22
CD07	300	8	Systemic allergic reaction at 2nd infusion, discontinuation
UC08	250	88	No remission achieved, variable disease course, therapy ongoing
UC09	150	96	Good response, remission, therapy ongoing
UC10	300	64	Total colectomy due to severe course of the disease 60 weeks after vedolizumab start
UC11	300	52	Good response, remission, therapy ongoing
IBD-U12	300	52	No remission achieved, variable disease course, therapy ongoing

In the CD group, the RTE was 0.56 (95% CI 0.36–0.70) at baseline, 0.46 (95% CI 0.32–0.62) at week 14, and 0.44 (95% CI 0.30–0.64) at week 38. In the UC group, the RTE was 0.75* at baseline and 0.25* (* estimated variance = 0, 95% CI cannot be calculated) at weeks 14 and 38.

### Nutritional status and laboratory values

Patients with CD showed a weight gain from 44.4 kg (range, 31.0–62.9 kg) at baseline to 48.0 kg (range, 29.4–58.2 kg) at week 38. Considering physiological age-depending weight gain, comparison of the mean Z- Score at the beginning of therapy and at week 38 showed an increase from 1.05 to 1.26. The median albumin level increased from 29.6 g/l (range, 18.0–37.5 g/l) to 35.0 g/l (range, 28.1–42.4 g/l). The median haemoglobin level increased from 8.4 g/dl (range, 6.9–13.0 g/dl) at baseline to 10.5 g/dl (range, 7.8–12.3 g/dl) at week 38.

Patients with UC showed a weight gain from 34.3 kg (range, 20.5–42.0 kg) at baseline to 44.0 kg (range, 24–47 kg) at week 38. The median albumin level increased from 27.9 g/l (range, 25.6–41.0 g/l) at baseline to 40 g/l (range, 36.5–48.0 g/l) at week 38. Mean Z- Score of the weight increased from 1.36 to 1.86. The median albumin level increased from 27.9 g/l (range, 25.6–41.0 g/l) at baseline to 40 g/l (range, 36.5–48.0 g/l) at week 38. The median haemoglobin level increased from 10.4 g/dl (range, 8.0–12.3 g/dl) at baseline to 12.1 g/dl (range, 8.0–14.8) at week 38. Median fecal calprotectin dropped significantly in the UC cohort.

For both groups, no significant changes were observed in height velocity, CRP and HKT levels (Table 2).

### Corticosteroids and concomitant medication

In the CD group, 4/6 patients had concomitant corticosteroid therapy at baseline, which could be tapered down, but not completely discontinued. In the UC cohort, 4/5 patients had corticosteroids at baseline, but the therapy could be discontinued in all cases before week 14, but had to be restarted in one patient before week 38. Dosage of corticosteroids and concomitant medication including immunomodulators and mesalazine at baseline and week 38 are shown in Table 2. In the UC cohort one patient had metronidazole at baseline, but not at week 38 and another patient had tacrolimus at both time points (dosage per body weight 0.1 mg vs. 0.08 mg).

### Adverse events

One patient discontinued therapy due to a severe general systemic allergic reaction with dyspnoea at the second infusion of vedolizumab. This patient had also shown a systemic reaction to infliximab and a local skin reaction to adalimumab. No other infusion reactions or serious adverse events were reported.

## Discussion

The present study adds to the scarce literature on vedolizumab use in paediatric IBD, and suggests that vedolizumab is safe and might provide a clinical benefit. Our cohort consisted of patients with refractory disease, in whom conventional therapy had failed. At week 14, clinical remission was achieved in 1/7 of the patients with CD and 4/5 of the patients with UC. Only one patient discontinued therapy due to a severe general systemic allergic reaction with dyspnoea at the second infusion of vedolizumab. These results are consistent with previous paediatric investigations that showed a significant reduction in activity scores only in patients with UC, with sample sizes of 64, 52, and 21, respectively [14–16].

At week 14, 4/5 patients with UC achieved clinical remission, which is comparable to the rates reported in Singh et al. and Conrad et al. (week 14 clinical remission, 13/17 and 3/4 respectively), but is higher than that reported in Ledder et al. (week 14 clinical remission rate, 16/34). Although a significant reduction in the PUCAI scores was observed by week 2, 4/5 patients had concomitant corticosteroid therapy at this time. At the last follow-up, two patients in the UC group were still in remission; one patient had to undergo a total colectomy due to a severe disease course and loss of response, despite shortening of the dosing interval; and two patients presented a variable disease course. These results are consistent with the corticosteroid-free remission rate of 16/41 in paediatric UC after 12 months of therapy reported in Ledder et al. [16]. Additionally, the colectomy rate during vedolizumab treatment is similar to that in all previous paediatric reports (1/5, 4/22 and 6/41 respectively) [14–16]. In previous reports, paediatric patients with CD had a poorer response to vedolizumab therapy than patients with UC [14–16]. In the present study, the patients in the CD cohort had longer and more severe disease courses compared to those in the UC cohort before the introduction of vedolizumab. This may contribute to the lower response rate in the patients with CD. Shelton et al. suggested that the transmural healing process in CD might take longer compared to that in UC [21]. In addition, TNF-α antagonists downregulate MAdCAM-1 expression; thus, patients with CD with prior anti-TNF exposure may require more time to respond to treatment [13, 22].

The RTE values in the CD cohort remained similar across the observation period, and were lower in weeks 14 (0.46) and 38 (0.44) compared to that at baseline (0.56). Even more pronounced, RTEs in the UC cohort dropped markedly from 0.75 at baseline to 0.25 in weeks 14 and 38. In conclusion, the RTE indicates improvements in disease activity scores in the UC cohort, but not in the CD cohort.

Regarding secondary outcomes, growth failure and nutritional status in IBD patients, especially in children and adolescents, is a big concern [23]. Particularly as body image concerns impact the quality of life in this cohort [24]. In our data substantial weight gain in UC and a trend in CD was observed, while in the largest paediatric cohort study on vedolizumab thus far, no improvement in height velocity and no significant weight gain were observed during treatment [16]. Height and weight were not presented in two other paediatric studies [14, 15].

An improvement in laboratory markers, such as albumin and haemoglobin, were observed in both groups, relative to baseline values. In previous paediatric studies, no relevant treatment-related changes were found in HKT, albumin, and CRP levels [14]. Similarly, Singh et al. reported a treatment-related decrease in CRP levels in patients with CD, while CRP values were normal at baseline in the patients with UC, and albumin and HKT levels were normal at baseline and did not change over time [15].

In the largest paediatric cohort study on vedolizumab thus far, no improvement in height velocity and no significant weight gain were observed during treatment, but a decrease in faecal calprotectin was observed at the last follow-up compared to that at baseline [16].

Regarding safety concerns and severe adverse events, one patient, who had previously shown a systemic reaction to infliximab and a local skin reaction to adalimumab, had a severe systemic allergic reaction at the second infusion of vedolizumab. No other infusion reactions or severe adverse events were observed. Consistent with these results, minor adverse events, but no severe adverse events or allergic reactions, were reported in the existing paediatric studies. Similarly, GEMINI I and II reported no severe adverse events in adult patients, while GEMINI III reported one (< 1%) drug-related severe adverse event [11, 13, 25].

The present study has some limitations. The study was retrospective in nature and the sample size was small, even though we included all paediatric patients who received vedolizumab in Austria from November 2014 to August 2016; thus, firm conclusions on the effect of vedolizumab cannot be drawn. In particular, it should be emphasized that the *p* values and the corresponding statements about statistical significance included in the present manuscript must not be over-interpreted, since the sample sizes are very limited. However, 8 of our patients were followed for at least 52 weeks, which is comparable to the study by Ledder et al. [16]. Due to the retrospective character of the study, it was not possible to include faecal calprotectin in the CD cohort and ESR values. Prospective long-term studies, like the currently enrolling VEDOKIDS trial are required to define the full safety profile and adequate dosing for vedolizumab treatment in children with IBD [22].

Our results suggest that vedolizumab may induce a clinical response and an improvement in soft markers, like weight gain. However, in children with a severe disease course or steroid-dependency, a loss of response to vedolizumab occurred quite often, and vedolizumab use was not successful in preventing a colectomy in one child with UC.

## Conclusions

In conclusion, the present study demonstrates that vedolizumab might be an effective treatment for individual paediatric patients with IBD who are unresponsive, intolerant, or experience a loss of efficacy in other therapies, including TNF-α antagonists. However, vedolizumab appears to be more effective in paediatric UC than in paediatric CD.

## Abbreviations

CD: Crohn’s disease
CRP: C-reactive protein
ESR: Erythrocyte sedimentation rate
HB: Haemoglobin
HKT: Haematocrit
IBD: Inflammatory bowel disease
IBD-U: Unclassified inflammatory bowel disease
PUCAI: Paediatric ulcerative colitis activity index
RTE: Relative Treatment Effect
shPCDAI: Short paediatric Crohn’s disease activity index
TNF- α: Tumour necrosis factor alpha
UC: Ulcerative colitis
